# Our Bureau of Information

**Published:** 1911-11-04

**Authors:** 


### OUR BUREAU OF INFORMATION.

### Practical Help for all Workers among the Sick.

MANY members of the medical profession, many clergymen, many superintendents, matrons, sisters, nurses, visitors, and workers of all kinds are often disturbed in mind for the want of the means to put them in the way of promptly dealing with a difficult case which may require special care or other help of a practical kind. They frequently do not know where to turn for the information they require, or how to procure the means without which the unfortunate object of their solicitude must suffer avoidable trials, and may even be deprived of the opportunity to contribute towards their own maintenance and comfort. The means are usually in existence. In these cases the difficulty arises from a want of intimate knowledge of all the agencies of every kind which are available to meet almost every kind of human need and disablement.

The time seems therefore to have come readily to make available our bureau of information, to which every worker may have free access through our columns by becoming a reader of THE HOSPITAL. If it is to prove practically useful we shall require the co-operation of our readers not only by making application to the Editor for help of all kinds when they need it, but by examining the cases and inquiries which we shall publish from week to week and sending whenever possible suggestions or offers of help in regard to particular cases mentioned on this page.

### HOW TO MAKE THE BUREAU A SUCCESS.

In order to facilitate as far as possible the work we have in view, everyone who uses or wishes to help our Bureau of Information must be kind enough to observe the following rules:—

(1) Postal replies cannot be given. Exception will only be made in very special cases at the Editor's discretion.

(2) Queries or answers relating to different cases must be written, or preferably typed, on separate sheets of paper, and the writing must never be crossed.

(3) Questions and replies received not later than Wednesday will, as far as possible, be dealt with in our issue of the following Saturday week.

(4) Letters must have attached the name and address of the sender, with a pseudonym for publication if preferred.

(5) *All communications* for this department *must* be *accompanied by the coupon* to be found at the bottom of the third page of the cover on the inside, and should be addressed to the Editor of the Bureau of Information, c/o THE HOSPITAL, 29 Southampton Street, Strand, London, W.C.

*Notice.*—We invite workers of special experience and knowledge to offer help in replying to questions relating to their special department.

Remember the object of the Bureau is to be practically helpful directly to workers amongst the sick, and indirectly to every sufferer who needs information and help. Knowledge saves time and shortens suffering. It helps the unfortunate, strengthens the worker, and tends to make us fuller men and women. We cannot all be Solomons, but by co-operation and intercommunication, being workers in the same field and imbued with the same desire to make this a brighter and happier world, if possible, together we can do many things which would be otherwise beyond our powers of accomplishment.

### Needs and Helpers.

### HOME WANTED FOR PARALYTIC MAN.

"NORTHAMPTON" writes: Can you tell me of a suitable nursing Home for my father, who is paralysed? He has now been in that condition for about sixteen months, and has been steadily improving all that time with massage and exercise on the Sandow system, but his speech (the stroke affected the right side) does not seem to make the improvement I could wish. He is quite able to look after himself so far as dressing and undressing is concerned, so that he requires very little attention. I cannot pay a great deal—at the outside £2 per week—so perhaps you will answer accordingly. Is there any difficulty in getting patients into these Homes?

### HOME WANTED FOR DEFECTIVE BOY.

"LATE HOSPITAL MATRON" writes: Can you tell me of a Home, near Worcester preferred, where a boy of nine years could be received for about £1 1s. per week. He has not walked or talked since birth, and cannot sit up without support.

### HOME FOR AN INVALID.

"MELROSE" writes: I write to ask if through your paper you should hear of a patient or invalid requiring a Home with nursing experience required. I wish to say that I have a very comfortable Home, and would be glad to receive anyone wanting the same. I have been a nurse for several years in London, and have good references should they be required. My husband is the male nurse at the infirmary, and we live opposite. It is very open and pleasant country, gravel soil, and considered healthy. Should you hear of any one I shall be much obliged to you for the same.

[We shall be pleased to forward any letters to the writer of the above, but we can take no responsibility in any way for the *bona fides*. Letters to be forwarded should be accompanied by a penny stamp.—ED., THE HOSPITAL.]

### WORK FOR CONSUMPTIVES IN SANATORIUMS.

"FORWARD" writes: I enclose coupon, and shall be obliged for information on the following matter: At what sanatorium have they in use for the patients a joiner's shop, what style of a building is used so as to be suitable for working in in all weathers, and what is the cost of building and equipping such?

### SOAP-AND-SUGAR POULTICE.

"M.B., C.M." writes: I should be much obliged if you could give me the scientific explanation of what is known as the "soap-and-sugar poultice." I read your paragraph in THE HOSPITAL, in this connection, some months ago. I am in the habit of using this method of treatment a great deal, and would like to have a reason for it, as some people, even doctors, are sceptical about it.

[Soap-and-sugar poultice is an old-fashioned home remedy which modern medical men were inclined to jeer at; but four or five years ago Sir Almroth Wright put forward a highly scientific explanation of its action.—ED. THE HOSPITAL.]

